# Laparoscopic Pancreaticoduodenectomy in Elderly Patients: Systematic Review and Meta-Analysis

**DOI:** 10.3389/fsurg.2022.807940

**Published:** 2022-03-04

**Authors:** Qiang Wang, Chengxin Chen, Haiyang Li

**Affiliations:** ^1^School of Clinical Medicine, Guizhou Medical University, Guiyang, China; ^2^Department of Hepatobiliary Surgery, The Affiliated Hospital of Guizhou Medical University, Guiyang, China

**Keywords:** laparoscopic pancreaticoduodenectomy, open pancreaticoduodenectomy, meta-analysis, systematic review, elderly

## Abstract

**Background:**

The safety and efficacy of laparoscopic pancreaticoduodenectomy (LPD) in elderly patients who often suffer from pre-existing conditions (e.g., cardiovascular diseases) and poor functional reserve remain unclear. This meta-analysis aimed to evaluate the safety and efficacy of LPD in elderly patients.

**Methods:**

A systematic literature search was conducted using the PubMed, Embase, Web of Science, and Cochrane Library databases. All studies published from their inception to January 2022 reporting perioperative outcomes after LPD in elderly patients were included in the search (Group 1, comparing the perioperative outcomes of LPD and OPD in elderly patients; Group 2, comparing the perioperative outcomes after LPD between elderly and non-elderly patients). The evaluated outcomes included perioperative mortality, postoperative complications, conversion, operative time, estimated blood loss (EBL), postoperative hospital stay (POHS), and readmission.

**Results:**

In total 8 studies were included in the meta-analysis. Pooled analysis of Group 1 showed that EBL, 90-day mortality, major morbidity, bile leak, POH, abdominal infection, reoperation, POP, POCE, and readmission were not significantly different between the LPD and the OPD group. LPD was associated with longer operative time, lower POPF rate, lower DEG rate, and shorter POHS. Pooled analysis of Group 2 showed that mortality, major morbidity, POPF, DEG, bile leak, POH, abdominal infection, reoperation, conversion, operative time, EBL, and readmission were not significantly different between the elderly and the non-elderly group. The POHS of elderly group was significantly longer than non-elderly group.

**Conclusion:**

LPD may be a safe and feasible procedure for elderly patients and is associated with short POHS.

## Introduction

According to the Global Health Observatory data released by the World Health Organization (WHO), in 2015, the average life expectancy of the global population was 71 years (1). As the life expectancy continues to increase, the number of elderly people continues to rise (2). Evidently, the risk of developing pancreatic cancer and other periampullary benign and malignant diseases increases with age (3–6). For pancreatic cancer patients, surgery remains the only treatment option enabling long-term survival (7). Hence, increasing life expectancy has led to more elderly patients requiring surgery, such as pancreaticoduodenectomy (PD).

PD which involves multiplex anatomical structures and requires extensive reconstruction. Consequently, it is one of the most challenging surgeries (8). Several studies demonstrated that PD could be implemented with admissible mortality and risk of complications in elderly patients, and age should not be a contraindication to open pancreaticoduodenectomy (OPD) (9, 10). Over the last decade, the enhancements in surgical technologies, developments in laparoscopic equipment, and progress of fast-track recovery theory have played key roles in the application of laparoscopic pancreaticoduodenectomy (LPD). In a systemic review, LPD has been confirmed as a safe and effective procedure based on shorter lengths of hospital stay, lower blood loss, and milder postoperative pain compared with OPD in selected patients (11). Nevertheless, LPD is a complex procedure, which requires long operative times and continuous pneumoperitoneum. Thus, despite LPD being performed more frequently in selected patients, the efficacy and safety of the procedure in elderly patients, who often suffer from pre-existing conditions (e.g., cardiovascular diseases) and poor functional reserve, remain unclear. Over the past few years, a number of studies (8, 12–15) have focused on the outcomes of LPD in elderly patients. However, to the best of our knowledge, few systematic reviews and meta-analyses evaluating this inconsistent issue. Thereby, we performed the present study to assess the safety and efficacy of LPD in elderly patients.

## Methods

This study was designed according to the Preferred Reporting Items for Systematic Reviews and Meta-Analyses (PRISMA) guidelines (16).

### Search Strategy

We conducted a systematic literature search using the PubMed, Embase, Web of Science, and Cochrane Library databases. All studies published from their inception to January 2022 reporting perioperative outcomes after LPD in elderly patients were included in the search. The following headings: “laparoscopy,” “laparoscopic,” “minimally invasive,” “Whipple's procedure,” “pancreaticoduodenectomy,” “elderly,” “geriatric,” “old,” “aged” were used in the advanced search. The key review articles and references of the retrieved studies were manually searched to discover further potentially relevant literature. In PubMed, the detailed literature search strategy is (“laparoscopy” [Title/Abstract] OR “laparoscopic” [Title/Abstract] OR “minimally invasive” [Title/Abstract]) AND (“elderly” [Title/Abstract] OR “geriatric” [Title/Abstract] OR “old” [Title/Abstract] OR “aged” [Title/Abstract]) AND (“Whipple's procedure” [Title/Abstract] OR “pancreaticoduodenectomy” [Title/Abstract]).

### Inclusion and Exclusion Criteria

Comparative studies on the effects of LPD in elderly patients were analyzed. The inclusion criteria in the light of the PICOS were defined as follows (16): (1) participants: elderly (≥ 70 years old) and non-elderly (<70 years old) patients suffered from pancreatic head and other periampullary benign and malignant tumors; (2) interventions and comparisons: (1) Group 1, comparing the perioperative outcomes of LPD and OPD in elderly patients, (2) Group 2, comparing the perioperative outcomes after LPD between elderly and non-elderly patients; (3) outcomes: perioperative mortality, postoperative complications [major morbidity, pancreatic fistula (POPF), delayed gastric emptying (DGE), bile leak, postoperative hemorrhage (POH), abdominal infection, reoperation, pneumonia (POP), cardiac events (POCE)], conversion, operative time, estimated blood loss (EBL), postoperative hospital stay (POHS), and readmission. (4) Study design: comparative studies. Case reports, review articles, commentaries, letters, conference abstracts, and studies with <10 patients were excluded. Additionally, studies that involved patients who underwent LPD and OPD not grouped by age (elderly and non-elderly) were excluded.

### Data Extraction and Quality Assessment

Two researchers independently evaluated the studies obtained from the above databases. If discrepancies emerged during the process of selection and evaluation, they were resolved by discussion or consultation with the third author. Two reviewers independently extracted and summarized material from each study. The collected information included: (1) The name of the first author, publication year, age and sex of the patients, and the number of patients; (2) study outcomes. Death within 90 days post-surgery was defined as perioperative mortality. Grade III or higher complication based on the Clavien–Dindo classification of surgical complications was defined as major morbidity (17). A POPF was defined based on the pancreatic fistula criteria of The International Study Group (18). The Newcastle-Ottawa Quality Assessment Scale (NOS) (19) was employed, which is typically used for evaluating and validating the quality of observational studies. Each study was awarded a score from 0 to 9 points. A study with a score of ≥6 was deemed high quality.

### Statistical Analysis

Review Manager version 5.3 (Nordic Cochrane Centre, Cochrane Collaboration, Copenhagen, Denmark) was utilized for statistical analysis. For dichotomous variables, the Mantel–Hansel method was used to estimate the odds ratio (OR) with a 95% confidence interval (CI). The inverse variance method was employed with a 95% CI when continuous parameters were reported as a mean and standard deviation. Heterogeneity was evaluated with the *I*^2^ statistic (20). *I*^2^ <30% was considered as low heterogeneity (21). A fixed-effects model was applied to calculate the pooled effects. 30% ≤ *I*^2^ ≤ 50% and *I*^2^ > 50% were considered as moderate and high heterogeneity, respectively (21). The pooled effects were calculated using a random-effects model when *I*^2^ ≥ 30%. *p* < 0.05 was accepted as indicative of significant differences in the review. If obvious heterogeneity was found, sensitivity analysis was conducted by excluding the studies one by one and recalculating the pooled OR and its 95% CI for the remaining studies to evaluate the stability of the results. If necessary, subgroup analyses were performed to elucidate obvious heterogeneity. Publication bias was evaluated by visual inspection of the funnel plot asymmetry, as previously described by Egger et al. (22).

## Results

### Search Results and Article Review

**Figure 1:**
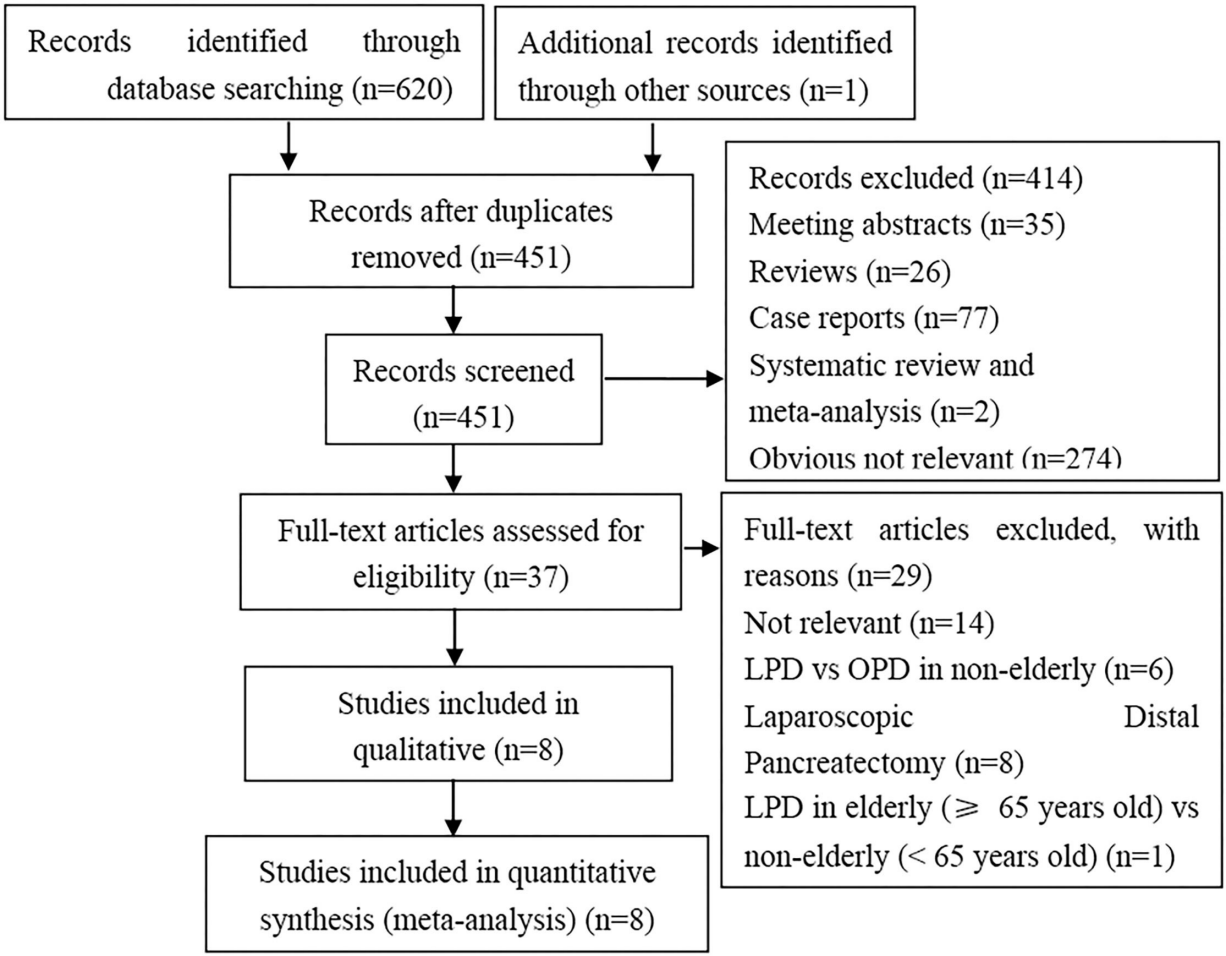
Flow diagram of the search method and selection process.

We retrieved 621 articles matching the initial search criteria. 170 duplicate articles were excluded. Among the remaining 451 articles, 414 were excluded following title and/or abstract screening according to the inclusion and exclusion criteria. Overall, after full-text review, 8 studies (8, 12–15, 23–25) were included in the meta-analysis. The process of selecting relevant studies is presented in Figure 1.

### Study Characteristics

#### Group 1: LPD vs. OPD in the Elderly

In total, 6 retrospective studies (8, 12–14, 24, 25) published between 2015 and 2021 were selected for the analysis. The cut-off age of the elderly population was 70 in four studies (8, 12, 14, 24), 75 in one study (13) and 80 in the remaining study (25). A total of 2,600 elderly patients, 519 and 2,081 of whom underwent LPD and OPD, respectively, were included in the meta-analysis. Four (8, 12, 14, 24) out of six studies were from a single center. The type of pathology included those works was distal common bile duct cancer, ampullary cancer, duodenal cancer, and other. Another study (25) was performed by two institutions of South Korea and the type of pathology was periampullary tumor. The remaining study (13) was conducted by the National Cancer Database of the USA and the type of pathology was restricted to pancreatic cancer. NOS showed that three studies (8, 12, 14) obtained a score of 7, while the remaining three studies (13, 24, 25) achieved a score of 6 (**Table 3**). The demographics of the study populations in group 1 are demonstrated in Table 1.

**Table 1:** Demographics of the study population (Group 1: LPD vs. OPD in elderly patients).

**Ref**.	**Approach**	**Age (years)**	***N* (%)**	**Gender (M/F), *n* (%)**	**PC/DC/A or D/other, *n* (%)**
Shin et al. (8)	LPD	74.8 ± 3.7	56 (17.2)	27 (48.2)/29 (51.8)	14 (25.0)/19 (33.9)/23 (41.1)/0 (0)
	OPD	74.6 ± 3.5	270 (82.3)	153 (56.7)/117 (41.3)	115 (42.6)/92 (34.1)/63 (23.3)/0 (0)
Tee et al. (12)	LPD	76.5 ± 4.3	113 (33.4)	51 (45.1)/62 (54.9)	53 (46.9)/4 (3.5)/13 (11.5)/43 (38.1)
	OPD	76.4 ± 4.5	225 (55.6)	140 (62.2)/85 (37.8)	121 (53.8)/15 (6.7)/42 (18.6)/47 (20.9)
Chapman et al. (13)	LPD	79.6 ± 3.5	248 (14.6)	132 (53.2)/116 (47.8)	248 (100)/0 (0)/0 (0)/0 (0)
	OPD	79.5 ± 3.4	1,520 (85.4)	721 (47.4)/799 (52.6)	1,520 (100)/0 (0)/0 (0)/0 (0)
Liang et al. (14)	LPD	74 ± 4	27 (58.7)	16 (59.3)/11 (40.7)	12 (44.4)/NA/12 (44.4)/3 (11.2)
	OPD	76 ± 5	19 (41.3)	13 (68.4)/6 (31.6)	15 (78.9)/NA/2 (10.5)/2 (10.5)
Tan et al. (24)	LPD	75.2 ± 4.4	56 (66.7)	33 (58.9)/23 (41.1)	21 (37.5)/13 (23.2)/10 (17.9)/12 (21.4)
	OPD	74.7 ± 4.6	28 (33.3)	16 (57.1)/12 (42.9)	11 (39.3)/4 (14.3)/7 (25.0)/6 (21.4)
Kim et al. (25)	LPD	81 ± 1.64	19 (50.0)	7 (36.8)/12 (63.2)	4 (21.1)/11 (57.9)/4 (21.1)/0 (0)
	OPD	81 ± 1.07	19 (50.0)	7 (36.8)/12 (63.2)	11 (57.9)/5 (26.3)/3 (15.8)/0 (0)

*LPD, laparoscopic pancreaticoduodenectomy; OPD, open pancreaticoduodenectomy; PC, pancreatic cancer; DC, distal cholangiocarcinoma; A or D, ampullary or duodenal cancer; Other, other pathology; NA, not available*.

#### Group 2: LPD in Elderly and Non-elderly Patients

In total, 4 retrospective studies (14, 15, 23, 24) from China were included in the analysis. 568 patients, who underwent LPD, were included in the analysis. 175 of them were elderly and 393 were non-elderly. The basic characteristics of the studies and patient demographics are summarized in Table 2. NOS showed that two studies (14, 23) obtained a score of 7, while the remaining two studies (15, 24) achieved a score of 6 (Table 3). The demographics of the study populations in group 2 are demonstrated in Table 4.

**Table 2:** Outcomes of Group 1 (LPD vs. OPD in elderly patients).

**Ref**.	**Approach**	**Mortality**	**Major**	**POPF**	**DEG**	**Bile leak**	**POH**	**Abdominal**	**Reoperation**	**POP**	**POCE**	**Operative time**	**EBL (ml)**	**POHS**	**Readmission**
			**morbidity *n* (%)**	***n* (%)**	***n* (%)**	***n* (%)**	***n* (%)**	**infection *n* (%)**	***n* (%)**	***n* (%)**	***n* (%)**	**(min)**		**(days)**	***n* (%)**
Shin et al. (8)	LPD	0 (0)	3 (5.4)	4 (7.1)	0 (0)	0 (0)	1 (1.8)	2 (3.6)	NA	0 (0)	0 (0)	321.8 ± 56.1	468.0 ± 331.0	13.5 ± 11.3	NA
	OPD	3 (1.1)	33 (12.2)	62 (23.0)	4 (1.5)	3 (1.1)	3 (1.1)	11 (4.1)	NA	6 (2.2)	6 (2.2)	268.5 ± 68.8	362.0 ± 363.0	16.5 ± 11.3	NA
Tee et al. (12)	LPD	5 (4.4)	11 (9.7)	26 (23.0)	27 (23.9)	NA	9 (8.0)	32 (28.3)	3 (2.7)	14 (12.4)	25 (22.1)	364.5 ± 110.6	344.7 ± 346.5	NA	19 (17.1)
	OPD	3 (1.3)	34 (15.1)	57 (25.3)	79 (35.1)	NA	19 (8.4)	68 (30.2)	15 (6.7)	33 (14.7)	56 (24.9)	359.8 ± 90.0	868.8 ± 1118.2	NA	37 (16.5)
Chapman et al. (13)	LPD	9 (3.6)	NA	NA	NA	NA	NA	NA	NA	NA	NA	NA	NA	NA	NA
	OPD	66 (4.3)	NA	NA	NA	NA	NA	NA	NA	NA	NA	NA	NA	NA	NA
Liang et al. (14)	LPD	2 (7.0)	11 (40.7)	4 (14.8)	0 (0)	NA	4 (14.8)	5 (18.5)	3 (11.1)	NA	NA	368.0 ± 75	200.0 ± 75.0	12 ± 2.75	2 (7)
	OPD	2 (10.0)	8 (42.1)	4 (21.1)	1 (5.3)	NA	0 (0)	6 (31.6)	1 (5.3)	NA	NA	369.0 ± 73	400.0 ± 125.0	18 ± 7.25	0 (0)
Tan et al. (24)	LPD	2 (3.6)	5 (8.9)	4 (14.8)	3 (5.4)	NA	2 (3.6)	NA	2 (3.6)	NA	NA	380.0	300.0	15.5	2 (3.6)
	OPD	2 (7.1)	5 (17.9)	4 (21.1)	4 (14.3)	NA	2 (7.1)	NA	2 (7.1)	NA	NA	292.5	250.0	18.0	1 (3.6)
Kim et al. (25)	LPD	0 (0)	2 (10.5)	2 (10.5)	2 (10.5)	0 (0)	1 (5.3)	0 (0)	NA	1 (5.3)	NA	441.0 ± 61.87	325.0 ± 198.78	NA	NA
	OPD	0 (0)	1 (5.3)	1 (5.3)	2 (10.5)	1 (5.3)	1 (5.3)	1 (5.3)	NA	1 (5.3)	NA	338.0 ± 53.01	518.0 ± 461.45	NA	NA

*LPD, laparoscopic pancreaticoduodenectomy; OPD, open pancreaticoduodenectomy; POPF, postoperative pancreatic fistula; DGE, delayed gastric emptying; POH, postoperative hemorrhage; POP, postoperative pneumonia; POCE, post-operative cardiac events; EBL, estimated blood loss; POHS, postoperative length of hospital stay; NA, not available*.

**Table 3:** Newcastle-Ottawa Scale (NOS) assessment of non-randomized studies.

**Study**	**Selection**			**Comparability**			**Outcome**			
	**1**	**2**	**3**	**4**	**5**	**6**	**7**	**8**	**9**	**Total**
Shin et al. (8)	✰	✰	✰	✰	✰		✰	✰		7
Tee et al. (12)	✰	✰	✰	✰	✰	✰	✰			7
Chapman et al. (13)	✰	✰	✰	✰			✰	✰		6
Liang et al. (14)	✰	✰	✰	✰	✰		✰	✰		7
Meng et al. (15)	✰	✰	✰	✰			✰	✰		6
Cai et al. (23)	✰	✰	✰	✰	✰		✰	✰		7
Tan et al. (24)	✰	✰	✰	✰			✰	✰		6
Kim et al. (25)	✰	✰	✰	✰			✰	✰		6

*1. Representativeness of exposed cohort; 2. Selection of non-exposed cohort; 3. Ascertainment of exposure; 4. Outcome of interest was not present at start of study; 5. Study controls for age, sex, and BMI; 6. Study controls for any additional factors; 7. Assessment of outcomes; 8. Follow-up long enough for outcomes to occur; 9. Adequacy of follow-up; ✰, 1 point*.

**Table 4:** Demographics of the study population (Group 2: LPD in elderly patients and non-elderly patients).

**Refs**.	**Age (years)**	***N* (%)**	**Gender, (M/F), *n* (%)**	**ASA (≥3), *n* (%)**	**PC/DC/A or D/other, *n* (%)**
Liang et al. (14)	Elderly: 74.0 ± 4.0	27 (33.0)	16 (59.3)/11 (40.7)	8 (30.0)	12 (44.4)/NA/12 (44.4)/NA
	Non-elderly: 59.0 ± 9.0	55 (67.0)	30 (54.5)/25 (45.5)	1 (1.8)	18 (32.7)/NA/17 (30.9)/NA
Meng et al. (15)	Elderly: 73.0	41 (20.6)	28 (68.3)/13 (31.7)	28 (68.3)	16 (39.0)/10 (24.4)/9 (22.0)/6 (14.6)
	Non-elderly: 56.5	158 (79.4)	97 (61.4)/61 (38.6)	99 (62.7)	34 (21.5)/24 (15.2)/44 (27.8)/56 (35.4)
Cai et al. (23)	Elderly: 75.2 ± 3.9	51 (34.7)	30 (58.8)/21 (41.2)	15 (29.4)	51 (100)/0 (0)/0 (0)/0 (0)
	Non-elderly: 56.1 ± 9.4	96 (65.3)	35 (36.5)/61 (63.5)	24 (25.0)	96 (100)/0 (0)/0 (0)/0 (0)
Tan et al. (24)	Elderly: 75.2 ± 4.4	84 (60.0)	33 (58.9)/24 (41.1)	19 (33.9)	21 (37.5)/13 (23.2)/10 (17.9)/12 (21.4)
	Non-elderly: 60.7 ± 7.5	56 (40.0)	51 (60.7)/33 (39.3)	18 (21.4)	22 (26.2)/20 (23.8)/14 (16.7)/28 (33.3)

*LPD, laparoscopic pancreaticoduodenectomy; ASA, American Society of Anesthesiologists classification; PC, pancreatic cancer; DC, distal cholangiocarcinoma; A or D: ampullary or duodenal cancer; Other, other pathology; NA, not available*.

### Outcomes

#### Group 1: LPD vs. OPD in the Elderly

**Figure 2:**
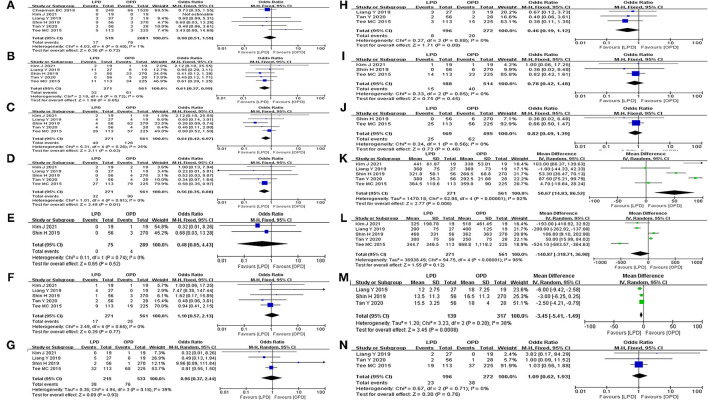
Forest map of group 1 (LPD vs. OPD in elderly patients). **(A)** Mortality. **(B)** Major morbidity. **(C)** POPF. **(D)** DEG. **(E)** Bile leak. **(F)** POH. **(G)** Abdominal infection. **(H)** Reoperation. **(I)** POP. **(J)** POCE. **(K)** Operative time. **(L)** EBL. **(M)** POHS. **(N)** Readmission.

Post-operative mortality was reported in six studies (8, 12–14, 24, 25). No statistical difference was found in 90-day mortality rate between the LPD and the OPD group (OR: 0.90, 95%CI = 0.51–1.59, *p* = 0.72). Major morbidity, POPF, DEG, POH, operative time, and EBL were reported in five studies. The results of the meta-analysis indicated that the major morbidity rate (OR: 0.61, 95%CI = 0.37–0.99, *p* = 0.05) and POH rate (OR: 1.10, 95%CI = 0.57–2.13, *p* = 0.77) between the LPD and the OPD group were not significantly different. There was no statistical difference in EBL (MD: −141.06 95%CI = −318.82 to 36.70, *p* = 0.12) between the LPD and the OPD group. However, the POPF rate (OR: 0.64, 95%CI = 0.42–0.97, *p* = 0.03) and DEG rate (OR: 0.56, 95%CI = 0.35–0.88, *p* = 0.01) in LPD group were significantly lower than OPD group. Additionally, the operative time of LPD group was significantly longer than OPD group (MD: 50.67 95%CI = 14.83–86.52, *p* = < 0.01). Abdominal infection was reported in four studies. No statistical difference was found in abdominal infection rate between the LPD and the OPD group (OR: 0.96, 95%CI = 0.37–2.44, *p* = 0.93). Reoperation, POP, POHS, and readmission were reported in three studies. No statistical difference was found in reoperation rate (OR: 0.46, 95%CI = 0.19–1.12, *p* = 0.09), POP rate (OR: 0.78, 95%CI = 0.42–1.48, *p* = 0.45), and readmission rate (OR: 1.09, 95%CI = 0.62–1.93, *p* = 0.76) between the LPD and the OPD group. However, the POHS of LPD group was significantly longer than OPD group (MD: −3.45 95%CI = −5.41 to −1.49, *p* = < 0.01). Bile leak and POCE were reported in two studies. There was no statistical difference in bile leak rate (OR: 0.48, 95%CI = 0.05–4.43, *p* = 0.52) and POCE rate (OR: 0.82, 95%CI = 0.49–1.39, *p* = 0.46) between the LPD and the OPD group. All outcomes between the LPD and the OPD group in the analyzed studies are shown in Table 2. The pooled outcomes of the meta-analysis are summarized in Table 5. All forest plots for Group 1 are shown in Figure 2.

**Table 5:** Pooled outcomes of meta-analysis of LPD vs. OPD in elderly patients.

**Outcomes**	**No. studies**	**Sample size**		**Heterogeneity(*****P***, ***I*****^2^)**		**Model**	**Overall effect size**	**95% CI**	***P***
		**(LPD vs. OPD)**							
Mortality	6	519	2081	0.40	1%	Fixed	OR = 0.90	0.51–1.59	0.72
Major morbidity	5	271	561	0.72	0%	Fixed	OR = 0.61	0.37–0.99	0.05
POPF	5	271	561	0.26	25%	Fixed	OR = 0.64	0.42–0.97	**0.03**
DEG	5	271	561	0.91	0%	Fixed	OR = 0.56	0.35–0.88	**0.01**
Bile leak	2	75	289	0.74	0%	Fixed	OR = 0.48	0.05–4.43	0.52
POH	5	271	561	0.65	0%	Fixed	OR = 1.10	0.57–2.13	0.77
Abdominal infection	4	215	533	0.18	39%	Random	OR = 0.96	0.37–2.44	0.93
Reoperation	3	196	271	0.88	0%	Fixed	OR = 0.46	0.19–1.12	0.09
POP	3	188	514	0.85	0%	Fixed	OR = 0.78	0.42–1.48	0.45
POCE	2	169	495	0.56	0%	Fixed	OR = 0.82	0.49–1.39	0.46
Operative time	5	271	561	<0.01	**92%**	Random	MD = 50.67	14.83–86.52	**<0.01**
EBL	5	271	561	<0.01	**96%**	Random	MD = −141.06	−318.82 to 36.70	0.12
POHS	3	139	371	0.20	38%	Random	MD = −3.45	−5.41 to −1.49	**<0.01**
Readmission	3	196	272	0.71	0%	Fixed	OR = 1.09	0.62–1.93	0.76

*EBL, estimated blood loss; POPF, postoperative pancreatic fistula; DGE, delayed gastric emptying; POH, postoperative hemorrhage; POP, postoperative pneumonia; POCE, postoperative cardiac events; POHS, postoperative length of hospital stay; LPD, laparoscopic pancreaticoduodenectomy; OPD, open pancreaticoduodenectomy; OR, odds ratio; MD, mean deviation; CI, confidence interval. Bold, significant heterogeneity (in Heterogeneity), statistical difference (in P value)*.

#### Group 2: LPD in Elderly Patients and Non-elderly Patients

**Figure 3:**
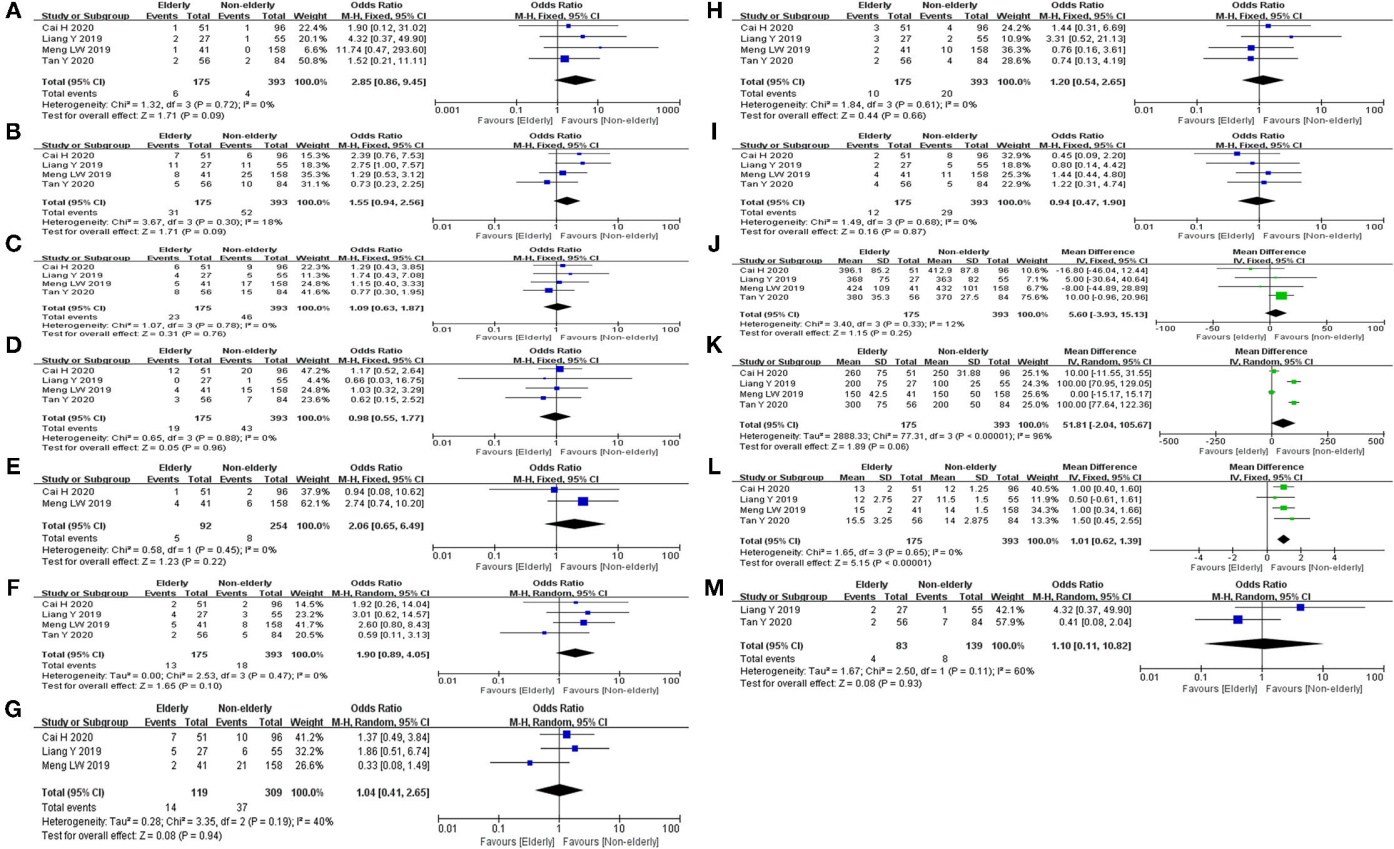
Forest map of group 2 (LPD in elderly patients and non-elderly patients). **(A)** Mortality. **(B)** Major morbidity. **(C)** POPF. **(D)** DEG. **(E)** Bile leak. **(F)** POH. **(G)** Abdominal infection. **(H)** Reoperation. **(I)** Conversion. **(J)** Operative time. **(K)** EBL. **(L)** POHS. **(M)** Readmission.

Mortality, major morbidity, POPF, DEG, POH, reoperation, conversion, operative time, EBL, and POHS were reported in four studies (14, 15, 23, 24). No statistical difference was found in mortality rate (OR: 2.85, 95%CI = 0.86–9.45, *p* = 0.09), major morbidity rate (OR: 1.55, 95%CI = 0.94–2.56, *p* = 0.09), POPF rate (OR: 1.09, 95%CI = 0.64–1.87, *p* = 0.97), DEG rate (OR: 0.98, 95%CI = 0.55–1.77, *p* = 0.96), POH rate (OR: 1.90, 95%CI = 0.89–4.05, *p* = 0.10), reoperation rate (OR: 1.20, 95%CI = 0.54–2.65, *p* = 0.66), and conversion rate (OR: 0.94, 95%CI = 0.47–1.90, *p* = 0.87) between the elderly and the non-elderly group. There were no statistical difference in operative time (MD: 5.60, 95%CI = −3.94 to 15.13, *p* = 0.25) and EBL (MD: 51.81, 95%CI = −2.04 to 105.67, *p* = 0.06) between the elderly group and the non-elderly group. However, the POHS of elderly group was significantly longer than non-elderly group (MD: 1.01, 95%CI = 0.62– 1.39, *p* ≤ 0.01). Abdominal infection was reported in three studies. No statistical difference was found in abdominal infection rate between the elderly group and the non-elderly group (OR: 1.04, 95%CI = 0.41–2.65, *p* = 0.94). POH and readmission were reported in two studies. There were no statistical difference in bile leak rate (OR: 2.06, 95%CI = 0.65–6.49, *p* = 0.22) and readmission rate (OR: 1.10, 95%CI = 0.11–10.82, *p* = 0.93) between the elderly group and the non-elderly group. All outcomes between the elderly and the non-elderly group in the analyzed studies are shown in Table 6. The pooled outcomes of the meta-analysis are shown in Table 7. All forest plots for Group 2 are shown in Figure 3.

**Table 6:** Outcomes of Group 2 (LPD in elderly patients and non-elderly patients).

**Refs**.	**Age**	**Mortality**	**Major**	**POPF**	**DGE**	**Bile leak**	**POH**	**Abdominal infection**	**Reoperation**	**POP**	**Conversion**	**Operative time**	**EBL (ml)**	**POHS**	**Readmission**
			**morbidity**	***n* (%)**	***n* (%)**	***n* (%)**	***n* (%)**	***n* (%)**	***n* (%)**	***n* (%)**	***n* (%)**	**(min)**		**(days)**	***n* (%)**
Liang et al. (14)	Elderly	2 (7)	11	4 (14.8)	0 (0)	NA	4 (14.8)	5 (18.5)	3 (11.1)	NA	2 (7.4)	368 ± 75	200.0	12 ± 2.75	2 (7)
	Non-elderly	1 (2)	11	5 (9.1)	1 (1.8)	NA	3 (5.5)	6 (10.9)	2 (3.6)	NA	5 (9.1)	363 ± 82	100.0	11.5 ± 1.5	1 (2)
Meng et al. (15)	Elderly	1 (2.4)	8	5 (12.2)	4 (9.8)	4 (9.8)	5 (12.2)	2 (4.9)	2 (4.9)	4 (9.8)	4 (9.8)	424 ± 109	150.0	15 ± 2	NA
	Non-elderly	0 (0)	25	17 (10.8)	15 (9.5)	6 (3.8)	8 (5.1)	21 (13.3)	10 (6.3)	8 (5.1)	11 (7.0)	432 ± 101	150.0	14 ± 1.5	NA
Cai et al. (23)	Elderly	1 (2.0)	7 (13.8)	6 (11.7)	12 (23.5)	1 (2.0)	2 (3.9)	7 (13.7)	3 (5.9)	NA	2 (3.9)	396.1 ± 85.2	260.0	13 (9.0–17.0)	NA
	Non-elderly	1 (1.0)	6 (6.2)	9 (9.3)	20 (20.8)	2 (2.1)	2 (4.2)	10 (10.4)	4 (4.2)	NA	8 (8.3)	412.9 ± 87.8	250.0	12 (10.0–15.0)	NA
Tan et al. (24)	Elderly	2 (3.6)	5 (8.9)	8 (14.3)	3 (5.4)	NA	2 (3.6)	NA	2 (3.6)	NA	4 (7.1)	380.0 (306.3–447.5)	300.0	15.5 (13.0–26.0)	2 (3.6)
	Non-elderly	2 (2.4)	10 (11.9)	15 (17.9)	7 (8.3)	NA	5 (6.0)	NA	4 (4.8)	NA	5 (6.0)	370.0 (310.0–420.0)	200.0	14.0 (11.3–22.8)	7 (8.3)

*LPD, laparoscopic pancreaticoduodenectomy; POPF, postoperative pancreatic fistula; DGE, delayed gastric emptying; POH, postoperative hemorrhage; POP, postoperative pneumonia; EBL, estimated blood loss; POHS, postoperative length of hospital stay; NA, not available*.

**Table 7:** Pooled outcomes of meta-analysis of LPD in elderly patients and non-elderly patients.

**Outcomes**	**No. studies**	**Sample size**		**Heterogeneity (*****P***, ***I*****^2^)**		**Model**	**Overall effect size**	**95% CI**	***P***
		**(elderly vs. non-elderly)**							
Mortality	4	175	393	0.72	0%	Fixed	OR = 2.85	0.86–9.45	0.09
Major morbidity	4	175	393	0.30	18%	Fixed	OR = 1.55	0.94–2.56	0.09
POPF	4	175	393	0.78	0%	Fixed	OR =9	0.64–1.87	0.76
DEG	4	175	393	0.88	0%	Fixed	OR = 0.98	0.55–1.77	0.96
Bile leak	2	92	254	0.45	0%	Fixed	OR = 2.06	0.65–6.49	0.22
POH	4	175	393	0.47	0%	Fixed	OR = 1.90	0.89–4.05	0.10
Abdominal infection	3	119	309	0.19	40%	Random	OR = 1.04	0.41–2.65	0.94
Reoperation	4	175	393	0.61	0%	Fixed	OR = 1.20	0.54–2.65	0.66
Conversion	4	175	393	0.68	0%	Fixed	OR = 0.94	0.47–1.90	0.87
Operative time	4	175	393	0.33	12%	Fixed	OR = 5.60	−3.93 to 15.13	0.25
EBL	4	175	393	<0.01	**96%**	Random	MD = 51.81	−2.04 to 105.67	0.06
POHS	4	175	393	0.65	0%	Fixed	MD = 1.01	0.62–1.39	**<0.01**
Readmission	2	83	139	0.11	**60%**	Random	OR = 1.10	0.11–10.82	0.93

*EBL, estimated blood loss; POPF, postoperative pancreatic fistula; DGE, delayed gastric emptying; POH, postoperative hemorrhage; POP, postoperative pneumonia; POCE, postoperative cardiac events; POHS, postoperative length of hospital stay; LPD, laparoscopic pancreaticoduodenectomy; OPD, open pancreaticoduodenectomy; OR, odds ratio; MD, mean deviation; CI, confidence interval. Bold, significant heterogeneity (in Heterogeneity), statistical difference (in P value)*.

### Sensitivity Analysis and Subgroup Analysis

**Figure 4:**
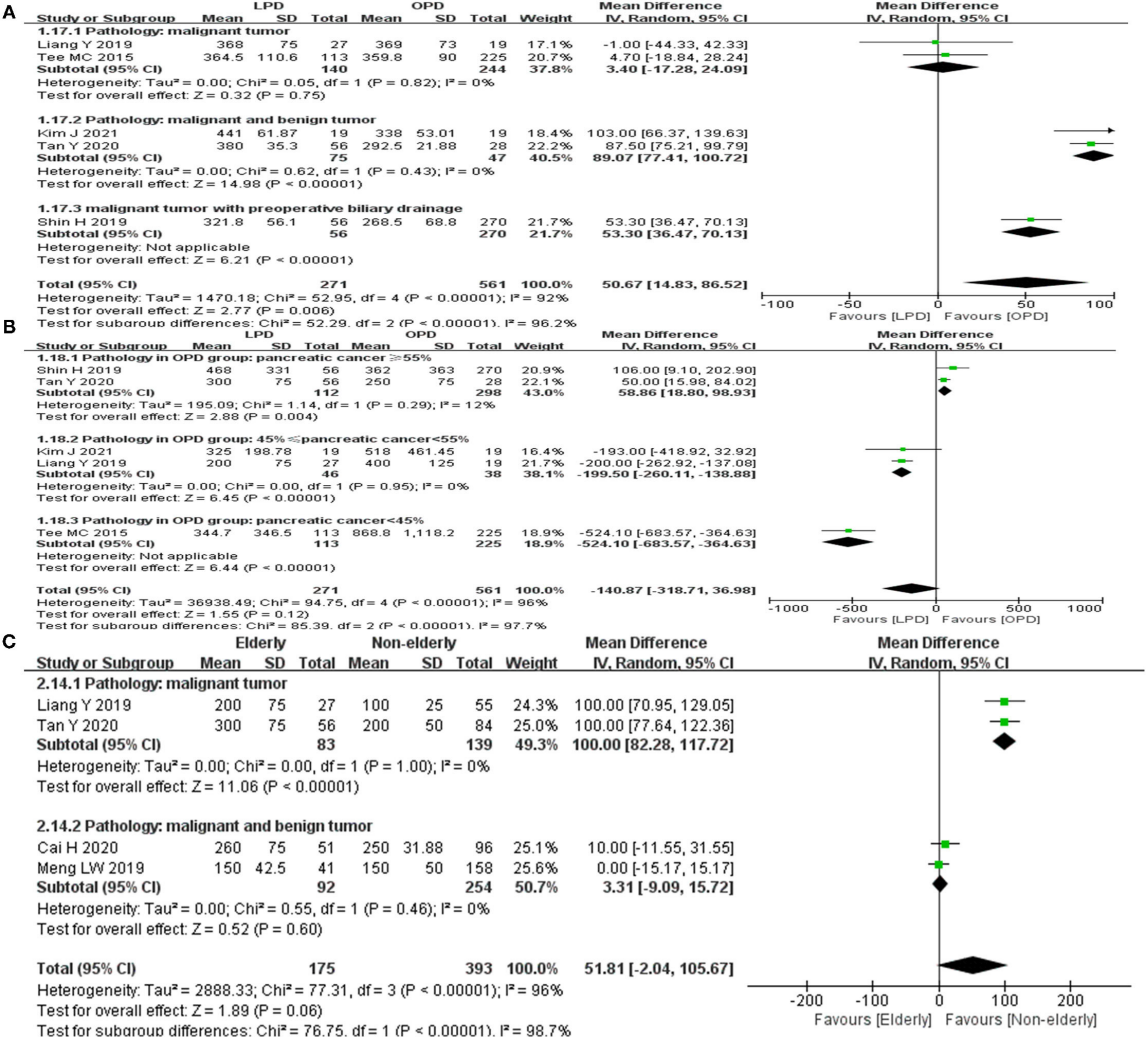
Subgroup analyses for group 1 and group 2. **(A)** Operative time in group 1. **(B)** EBL in group 1. **(C)** EBL in group 2.

In the meta-analysis of group 1, significant heterogeneity was found for the operative time (*I*^2^= 92%, *p* < 0.01) and EBL (*I*^2^= 96%, *p* < 0.01). In the sensitivity analysis, the recalculated MD of operative time ranged from 38.79 (95%CI = −1.70–79.27, *p* = 0.06) to 63.65 (95%CI = 31.57–75.73, *p* = 0.001). The recalculated MD of EBL ranged from −208.10 (95%CI = −428.40 to 12.20, *p* = 0.06) to −48.69 (95%CI = −208.08 to 110.71, *p* = 0.55). Sensitivity analysis revealed that the meta-analysis result for operative time was unstable. Subgroup analysis for the operative time indicated that the difference of pathological types may be the main reason for the high heterogeneity (Figure 4A). Sensitivity analysis revealed that the meta-analysis result for EBL was stable. Subgroup analysis for the EBL indicated that the difference of pathological types may be the main reason for the high heterogeneity (Figure 4B).

In the meta-analysis of group 2, significant heterogeneity was found for the EBL (*I*^2^= 96%, *p* < 0.01) and readmission rate (*I*^2^= 60%, *p* < 0.11). In the sensitivity analysis, the recalculated MD of EBL ranged from 35.37 (95%CI = −17.18 to 87.92, *p* = 0.19) to 69.64 (95%CI = 7.50–131.79, *p* = 0.03). The outcome of sensitivity analysis revealed that the meta-analysis results for EBL was unstable. For the readmission, sensitivity analysis and subgroup analysis could not be performed because of the limited number (*n* = 2) of included studies. Subgroup analysis for the EBL indicated that the difference of pathological types may be the main reason for the high heterogeneity (Figure 4C).

### Assessment Publication Bias

For group 1, only six studies (<10) were included in the meta-analysis. Similarly, just four studies were included in group 2. Hence, the publication bias was not assessed using a funnel plot.

## Discussion

Since 2004, the annual pancreatic cancer and pancreatic cancer-related mortality has increased by 1.5 and 0.5% among the American population, respectively (26). The data of the American Cancer Society showed that the 1-year and 5-year survival rates between 2001 and 2007 were 26 and 6%. Thus, at present, pancreatic cancer is one of the most sinister carcinomas. LPD is an effective method for treating pancreatic cancer. It has been frequently performed as a result of innovations in laparoscopic techniques and apparatus (27). A recent systematic review and meta-analysis showed that LPD was associated with reduced intraoperative bleeding, shortened LOHS, and comparable incidence of complications to OPD (28). Another systematic review and meta-analysis revealed fewer postoperative complications of LPD compared with OPD (29). One recent study reported by Zhang et al. (30) investigated the safety and efficacy of LPD in elderly patients. However, there was no clear definition of elderly patients in this study. In the comparison of elderly vs. non-elderly, elderly group included patients aged over 65 and 70, which could cause confusions. Consequently, the safety and efficacy evaluation may be underpowered. Therefore, it is still uncertain whether LPD is applicable to elderly patients.

In the present study, we followed strict inclusion and exclusion criteria. The safety and efficacy of LPD in elderly patients were comprehensively investigated. At last, we pooled six studies to compare the perioperative clinical outcomes of LPD (519 patients) and OPD (2,081 patients) in elderly patients. Concurrently, we pooled four studies to compare the perioperative outcomes of LPD in elderly (175) and non-elderly (393) patients. In the two studies reported by Liang et al. (14) and Tan et al. (24), the patients were divided into three groups (A: age <70 and underwent LPD, B: age ≥ 70 and underwent LPD, C: age ≥ 70 years and underwent OPD). In our meta-analysis, these two studies were included in both group 1 and group 2. In group 1, the pooled results illustrated that the mortality, major morbidity, bile leak, POH, abdominal infection, reoperation, POP, POCE, EBL, and readmission between the LPD and the OPD group in elderly patients were not significantly different.

The pooled result for EBL was inconsistent with the findings of several recent meta-analyses (28, 31, 32) which indicated lower EBL in LPD compared with OPD. In our meta-analysis, although LPD is a minimally invasive surgery, the longer operative time in LPD group may result in the same blood loss as OPD group. In addition, there was no statistical difference in the proportion of pancreatic cancer, 38.4% in LPD group and 48.7% in OPD group respectively. Evidently, the procedural complexities for pancreatic cancer such as R0 resection and lymph node dissection may cause LPD to loss blood equal to OPD. Although obvious heterogeneity was found, the sensitivity analysis revealed that the pooled result for EBL was stable. On the basis of subgroup analysis, the difference of pathological types (the proportion of pancreatic cancer) may be the main reason for the obvious heterogeneity.

Longer operative time was found in the LPD group than in the OPD group. The difference in pathology type may be one of the reasons for longer operative time in LPD group. The pathological types in two included studies (12, 14) were malignant tumors, and no difference in operative time was found in these two studies between the LPD and the OPD group. However, The pathological types of two other studies (24, 25) were malignant and benign tumors, and longer operative time was found in these two studies for LPD. Although laparoscopy can magnify the field of view, the procedural complexities such as the exposure of retroperitoneal space and the dissection of major vasculature are time-consuming. In addition, the experience of the surgeon such as controlling bleeding during laparoscopic surgery also affect the operative time. However, in all included studies, the experience of the surgeon was unclear, and it was not illustrated whether the surgical team has completed the learning curve. According to the sensitivity analysis, the pooled result for operative time was unstable. Subgroup analysis indicated that the difference in pathology type may be the main reason for unstable result and heterogeneity. Additionally, the study performed by Shin et al. (8) included a propensity score-matching analysis, while other works were retrospective cohort studies. This could be another cause of heterogeneity. At last, the different learning curves and experiences of surgical teams may result in unstable outcomes.

The conducted pooled analysis showed that the POPF rate and DGE rate in LPD group was lower than that in OPD group. This was inconsistent with the results of two recent meta-analyses (31, 32) which illustrated no significant differences in the POPF rate and DEG rate between LPD group and OPD group. POPF with severe clinical consequences is one of the most common complications of PD. The occurrence of POPF could affect postoperative recovery and mortality (33). DGE is another most common complication of PD. There are two plausible explanations for this discrepancy: (1) less interference with the gastrointestinal tract during LPD and (2) faster recovery of the gastrointestinal function due to milder postoperative pain. The lower POPF rate and DEG rate mean that LPD may be one of the suitable choice for elderly patients. Our study revealed that POHS in the LPD group was shorter than in the OPD group. This was consistent with the findings of several recent meta-analyses (28, 31, 32, 34). The shortened POHS means quick recovery and less cost in the whole treatment process, which may be one of the advantage of LPD in elderly patients. In general, although the operative time was longer in the LPD group, it was associated with shorter POHS, lower rate of POPH and DEG compared with the OPD group. This means that LPD for elderly patients may be as safe and feasible as OPD, or even superior to OPD to some extent.

As demonstrated in a previous meta-analysis, the incidence of postoperative major morbidity of PD between elderly (≥75) and non-elderly group was not significantly different (26). In group 2, our study revealed that the postoperative major morbidity rate of LPD between the elderly and the non-elderly group was also not significantly different. The conducted pooled analysis showed that the rate of POPF, POH, and reoperation for LPD was not significantly different between the elderly and the non-elderly group. This was consistent with the findings of several previous meta-analyses (28, 31, 32, 34). We found that the rate of mortality, DEG, bile leak, abdominal infection, conversion, and readmission of LPD was also not significantly different between the elderly and the non-elderly group. Our study did not determine any significant differences in operative time and EBL between the elderly and the non-elderly group. But the sensitivity analysis revealed that the pooled result for EBL was unstable. On the basis of subgroup analysis, the difference in pathological type may be also the main reason for the obvious heterogeneity. In our work, elderly patients was associated with longer POHS compared with non-elderly patients. However, it is not difficult to understand that poor physical functional status and slow postoperative recovery in elderly patients may lead to longer hospital stays. It is also possible that longer POHS could be caused by various external factors, such as the surgeon and patient preference, rather than complications (35). In general, various complications for LPD were not significantly different between the elderly and the non-elderly group, although the POHS was longer in the elderly group. This means that LPD for elderly patients may be as safe and feasible as non-elderly patients.

Certainly, our study had some limitations. Firstly, all studies included in our analysis were retrospective. Secondly, the difference between the pre-operative pathology type increased the risk of selection bias. Thirdly, the studies were from different medical centers, at which surgeons had varying operational skills, experience, and learning curve, resulting in bias. Moreover, a small number of studies with a small sample size were included in this work, which impeded heterogeneity testing and might cause inherent biases. We also did not assess the overall survival of elderly patients after LPD due to limited availability of data. Lastly, publication bias, which could influence the reliability of our results, cannot be fully excluded in this meta-analysis. Thus, the findings reported herein should be interpreted with caution. Well-designed randomized trials with a large sample size are necessary to further confirm our conclusions.

## Conclusion

The conducted meta-analysis revealed that LPD may be a safe and feasible procedure for elderly patients and is associated with short POHS. Age itself may not affect the postoperative mortality and complications and should not be considered as a limiting factor for LPD.

## Data Availability Statement

The original contributions presented in the study are included in the article/supplementary material, further inquiries can be directed to the corresponding author/s.

## Author Contributions

QW is the principal investigator with overall responsibility for the original draft. QW and CC performed searching for relevant studies, data collection, and data analysis. HL provided help in designing, data analysis and editing of the manuscript. All authors read and approved the final manuscript.

## Funding

This work is supported by the Science and Technology Program of Guizhou Province (No. [2020]4Y232).

## Conflict of Interest

The authors declare that the research was conducted in the absence of any commercial or financial relationships that could be construed as a potential conflict of interest.

## Publisher's Note

All claims expressed in this article are solely those of the authors and do not necessarily represent those of their affiliated organizations, or those of the publisher, the editors and the reviewers. Any product that may be evaluated in this article, or claim that may be made by its manufacturer, is not guaranteed or endorsed by the publisher.

## Abbreviations

LPD: laparoscopic pancreaticoduodenectomy
OPD: open pancreaticoduodenectomy
EBL: estimated blood loss
POPF: postoperative pancreatic fistula
DGE: delayed gastric emptying
POH: postoperative hemorrhage
POP: postoperative pneumonia
POCE: postoperative cardiac events
POHS: postoperative length of hospital stay
POPF: postoperative pancreatic fistula
DGE: delayed gastric emptying
POH: postoperative hemorrhage
POP: postoperative pneumonia
OR: odds ratio
MD: mean deviation
CI: confidence interval.
